# Dissecting Systemic RNA Interference in the Red Flour Beetle *Tribolium castaneum*: Parameters Affecting the Efficiency of RNAi

**DOI:** 10.1371/journal.pone.0047431

**Published:** 2012-10-25

**Authors:** Sherry C. Miller, Keita Miyata, Susan J. Brown, Yoshinori Tomoyasu

**Affiliations:** 1 Division of Biology, Kansas State University, Manhattan, Kansas, United States of America; 2 K-State Arthropod Genomics Center, Kansas State University, Manhattan, Kansas, United States of America; 3 Department of Zoology, Miami University, Oxford, Ohio, United States of America; Oxford Brookes University, United Kingdom

## Abstract

The phenomenon of RNAi, in which the introduction of dsRNA into a cell triggers the destruction of the corresponding mRNA resulting in a gene silencing effect, is conserved across a wide array of plant and animal phyla. However, the mechanism by which the dsRNA enters a cell, allowing the RNAi effect to occur throughout a multicellular organism (systemic RNAi), has only been studied extensively in certain plants and the nematode *Caenorhabditis elegans*. In recent years, RNAi has become a popular reverse genetic technique for gene silencing in many organisms. Although many RNAi techniques in non-traditional model organisms rely on the systemic nature of RNAi, little has been done to analyze the parameters required to obtain a robust systemic RNAi response. The data provided here show that the concentration and length of dsRNA have profound effects on the efficacy of the RNAi response both in regard to initial efficiency and duration of the effect in *Tribolium castaneum*. In addition, our analyses using a series of short dsRNAs and chimeric dsRNA provide evidence that dsRNA cellular uptake (and not the RNAi response itself) is the major step affected by dsRNA size in *Tribolium*. We also demonstrate that competitive inhibition of dsRNA can occur when multiple dsRNAs are injected together, influencing the effectiveness of RNAi. These data provide specific information essential to the design and implementation of RNAi based studies, and may provide insight into the molecular basis of the systemic RNAi response in insects.

## Introduction

RNA interference (RNAi) is a mechanism of gene silencing triggered by double-stranded RNA (dsRNA) [1]–[3]. dsRNA can induce gene silencing both at a post-transcriptional level (via mRNA cleavage and/or antisense suppression) and at a transcriptional level (through DNA modification) [3], [4]. While the RNAi pathway is an endogenous pathway known to be involved in regulating eukaryotic gene expression, defense against virus infection, and controlling transposon activity, it has also been harnessed as a reverse-genetic tool to inhibit gene expression in many organisms. The post-transcriptional RNAi pathway is initiated by the RNaseIII nuclease Dicer, which cleaves dsRNA into 21–23 bp fragments termed short interfering RNAs (siRNA) [5]–[7]. The siRNAs are then bound by a complex of proteins known as the RNA induced silencing complex (RISC) [8], [9]. This complex binds mRNA complementary to the siRNA and causes mRNA cleavage through the action of the catalytic Argonaute proteins [10]–[13]. The cleavage of mRNA reduces the amount of mRNA available for translation and thus phenocopies a loss of function mutation.

The RNAi phenomenon has been described and used as a genetic tool in classical genetic model organisms for over a decade. More recently, there has been a barrage of publications highlighting RNAi as an effective tool in many emerging model systems as well (for example, see [14]–[23]). However, many studies in these emerging model systems are limited in scope, with most of the data illustrating an RNAi effect for a limited number of genes, in specific tissues, at particular life stages. Because RNAi is a relatively new tool and has limited uses in *Drosophila* [24], there have been few investigations into the parameters required to make RNAi successful in insects. Furthermore, use of RNAi in mammals is difficult due to the interferon response, which can be trigged by dsRNA and can result in cell death [25]. Therefore, the vast majority of data available for the proper design of RNAi experiments is from one animal model system, the nematode *Caenorhabditis elegans*. The aim of this work is to provide information helpful for the experimental design of RNAi projects in *Tribolium castaneum* and other insects. Our results show that the size and concentration of dsRNA are critical to the effectiveness of the RNAi response, with longer dsRNA being more effective with respect to initial knockdown and duration of the RNAi effect. In addition, our analyses using a series of short dsRNAs and chimeric dsRNA provide evidence that dsRNA cellular uptake (and not the RNAi response itself) is the major step affected by dsRNA length in *Tribolium*. We also find that when multiple dsRNAs are injected, competition between dsRNAs can occur, resulting in a less effective RNAi response. The study of these basic features of RNAi in *Tribolium* will not only aid in experimental design but will also provide insight into the molecular mechanism of the systemic RNAi response in the red flour beetle.

## Results

### dsRNA Size

For RNAi experiments, the length of dsRNA used varies widely depending on the context and model organism (generally ranging from 21 to 1,000 base pairs). In plants, dsRNA length does not cause much difference in the RNAi efficiency, as both siRNAs and long dsRNAs have been shown to spread, silencing gene function both locally and systemically [26]. In animals, both siRNAs and long dsRNAs seem able to trigger the RNAi response if these RNA molecules are delivered directly inside the cells (via injection, transfection, and hair-pin construct overexpression) [27]. An exception to this is seen in mammalian cells, in which long dsRNAs induce the interferon response resulting in cell death [25]. The length of dsRNA molecules affects the efficiency of RNAi more prominently when dsRNAs are placed outside the cells in animals. In the nematode *C. elegans*, small dsRNAs and siRNAs are not efficiently taken up by cells and are insufficient to cause systemic RNAi [10], [28]–[31]. Therefore, long dsRNAs are required in order to induce a systemic RNAi response (the minimum length for efficient systemic RNAi in *C. elegans* is between 50 and 100 bp) [30], [31]. In insects, little is known about the dsRNA length requirements. Although *Drosophila* does not show a robust systemic RNAi response [24], *Drosophila* S2 cells (which possess hemocytes-like features) can efficiently take up long dsRNAs [32]. However, short dsRNA and siRNAs are not taken up in S2 cells without the aid of a transfection reagent [33], which reflects the similar tendency in *C. elegans*. In insects other than in the S2 cell culture condition, the size range of effective dsRNA has not been fully investigated. Most reports of RNAi use long dsRNA, although there have been limited reports of successful siRNA use in insects (including the pea aphid and the termite) [34], [35].

**Figure 1:**
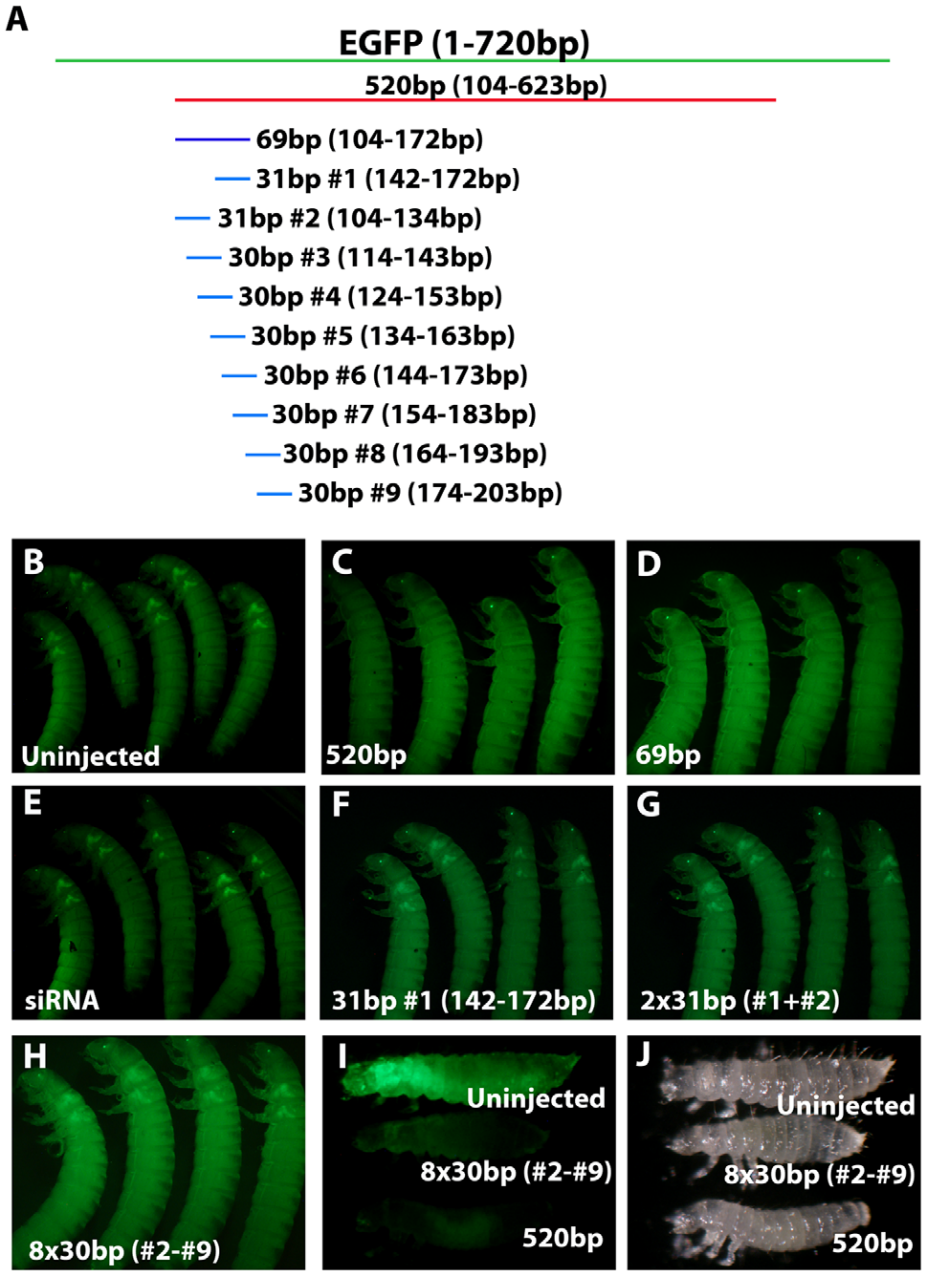
The effect of dsRNA size on RNAi knockdown efficiency. (A) Location of *EGFP* dsRNAs relative to the *EGFP* gene. Green: the full-length *EGFP* gene (minus noncoding portion). Red: long dsRNA (520 bp). Purple: intermediate dsRNA (69 bp). Blue: short dsRNA (30/31 bp). (B) Uninjected pu11 *Tribolium* larvae. (C-H) pu11 *Tribolium* larvae injected with *EGFP* dsRNA or siRNA. (C) 520 bp dsRNA. (D) 69 bp dsRNA. (E) 21 bp siRNA. (F) 31 bp (142–172 bp) dsRNA. (G) Two 31 bp dsRNAs. (H) Eight ∼30 bp dsRNAs. (I,J) pu11 *Tribolium* larvae injected as embryos with *EGFP* dsRNA. (I) Top larvae; uninjected, Middle larvae; injected with eight ∼30 bp dsRNAs, Bottom larvae; injected with 520 bp dsRNA. (J) Light microscopy image of (I).

To test the size requirements for dsRNA in *Tribolium,* we injected long dsRNAs or siRNAs corresponding to *EGFP* into the pu11 transgenic line [15], [36]. pu11 expresses an EGFP variant in the nervous system of first larval instars, in the wing discs and the larval eyes at the last larval stage, and in the eyes and wings at the pupal stage (Figure 1 B. Also see EGFP fluorescence analysis section in Methods). When long dsRNA (520 bp) was injected into last instar larvae, efficient knockdown of EGFP was seen in 100% of individuals at both the larval and pupal stages (n = 29) (Figure 1 C, Table S1. Data for pupal stages not shown). However, when a siRNA (21 bp) (silencer RNA, Ambion) corresponding to *EGFP* was used, normal levels of EGFP expression were observed in every injected individual (n = 28) (Figure 1 E, Table S1), suggesting that the siRNA is insufficient to induce a systemic RNAi response at the larval stage in *Tribolium*.

To further define the length of dsRNA necessary to achieve efficient EGFP knockdown, we synthesized an intermediate length dsRNA (69 bp) and a short dsRNA (31 bp). Our results indicate that the 69 bp fragment was efficient in knocking down EGFP in all individuals (n = 8) (Figure 1 D, Table S1) while the 31 bp fragment was not (n = 20) (Figure 1 F, Table S1). The results of the siRNA and 31 bp dsRNA injections suggest that short dsRNAs are not efficiently recognized by the dsRNA cellular uptake machinery and are therefore not readily incorporated into the cell, causing a weaker systemic RNAi response. However, it is known that not all siRNAs are equally efficient [37], [38], therefore it may be the specific sequence rather than the size that caused a difference in the efficiency of the RNAi response.

To determine whether the lack of sequence variety caused the short dsRNAs to be ineffective, we increased the sequence variety by synthesizing a second 31 bp dsRNA and coinjecting the two 31 bp dsRNA fragments. Together, the two 31 bp dsRNAs cover almost the entire region of *EGFP* targeted by the effective 69 bp fragment (Figure 1 A). However, these two fragments were also incapable of knocking down EGFP expression, as green fluorescence was still seen in 100% of individuals (n = 19) (Figure 1 G, Table S1). To further increase the sequence variety, we synthesized eight overlapping ∼30 bp dsRNAs spanning 100 bp of the *EGFP* coding region (Figure 1 A). In this way, we were able to drastically increase the sequence variety without increasing the length of the dsRNA. When these eight ∼30 bp dsRNA fragments were injected into *Tribolium* larvae (n = 21) they were still unable to knock down EGFP (Figure 1 H, Table S1), suggesting that it is not the lack of sequence variety that is causing the ineffectiveness. To determine whether the ineffective RNAi response was due to inefficient uptake by the cells, we injected the eight ∼30 bp fragments into *Tribolium* embryos at the syncytial blastoderm stage. At this stage of embryogenesis cell membranes have not yet formed around the multiple nuclei and therefore the dsRNA is being injected directly into a cell. After the larvae hatched from the eggs they were monitored for EGFP expression in the nervous system. We found that the eight ∼30 bp fragments were capable of knocking down EGFP expression when injected directly into the egg, as 89% of the hatched larvae showed no EGFP expression (n = 16) (Figure 1 I, Table S1). These data support the idea that small dsRNAs are ineffective at multicellular stages in *Tribolium* because they are unable to be taken up by the cells.

### dsRNA Concentration

**Figure 2:**
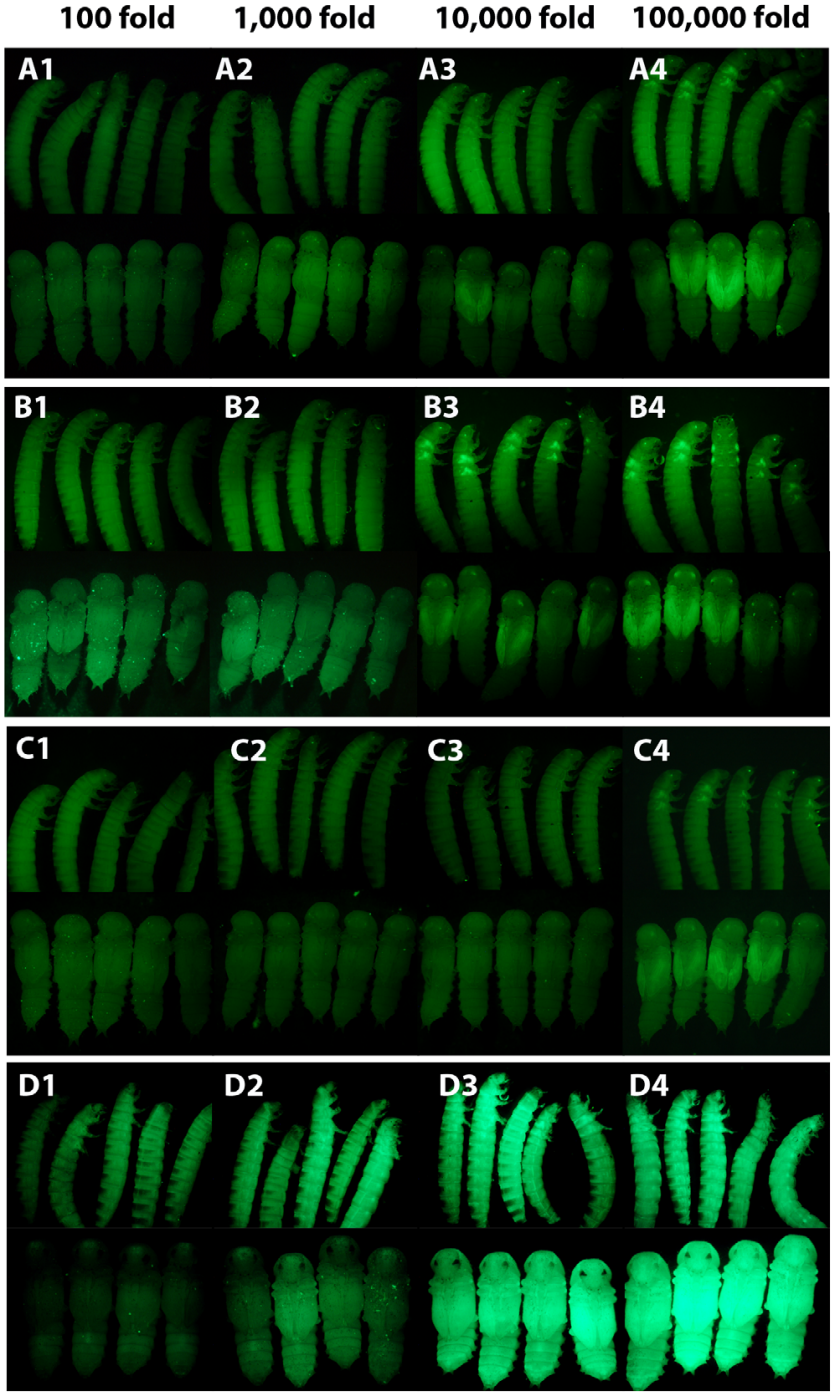
The effect of dsRNA concentration on RNAi knockdown efficiency. (A) Weight dilution series of 520 bp *EGFP* dsRNA injected into pu11 *Tribolium* larvae. (1) 0.01 µg/µL. (2) 0.001 µg/µL. (3) 0.0001 µg/µL. (4) 0.00001 µg/µL. (B) Weight dilution series of 69 bp *EGFP* dsRNA injected into pu11 *Tribolium* larvae. (1) 0.01 µg/µL. (2) 0.001 µg/µL. (3) 0.0001 µg/µL. (4) 0.00001 µg/µL. (C) Molar dilution series of 520 bp *EGFP* dsRNA injected into pu11 *Tribolium* larvae. (1) ∼0.07 µg/µL. (2) ∼0.007 µg/µL. (3) ∼0.0007 µg/µL (4) ∼0.00007 µg/µL. (D) Weight dilution series of 520 bp *EGFP* dsRNA injected into a tubulin EGFP *Tribolium* larvae. (1) 0.01 µg/µL. (2) 0.001 µg/µL. (3) 0.0001 µg/µL. (4) 0.00001 µg/µL.

To determine what concentrations of dsRNA are effective in *Tribolium*, we performed a serial dilution (from 1 µg/µL) of *EGFP* dsRNA (520 bp) and injected the dsRNA into pu11 larvae (Figure 2 A). We saw a complete absence of EGFP expression in all individuals injected at concentrations as low as 0.001 µg/µL (1,000 fold dilution) (Figure 2 A1, A2, Table S2). At a concentration of 0.0001 µg/µL (10,000 fold dilution), EGFP expression was reduced but still visible (Figure 2 A3, Table S2), and at a concentration of 0.00001 µg/µL (100,000 fold dilution) EGFP expression appeared comparable to wild-type levels (Figure 2 A4, Table S2).

Next we asked whether the length of the dsRNA injected influences the effectiveness of EGFP knockdown at low concentrations. We performed a serial dilution (from 1 µg/µL) of *EGFP* dsRNA (69 bp) (Figure 2 B), as this was the shortest size dsRNA we tested that worked efficiently at a higher concentration. As seen with the 520 bp dsRNA, the 69 bp dsRNA resulted in complete absence of EGFP expression in most individuals at concentration as low as 0.001 µg/µL (1,000 fold dilution) (Figure 2 B1, B2, Table S2). However, at 0.0001 µg/µL (10,000 fold dilution), EGFP expression in 100% of individuals appeared comparable to normal levels (Figure 2 B3, Table S2). This suggests that the 69 bp dsRNA may be slightly less effective than the longer 520 bp fragment, although finer scale dilutions need to be performed to determine at what concentration the 69 bp fragment begins to lose effectiveness.

The dilutions described above were calculated based on dsRNA weight. Longer dsRNA weighs more per molecule than shorter dsRNA. Therefore, when serial dilutions are based on weight, the dilutions of longer dsRNA will have fewer initial dsRNA molecules than dilutions of the shorter dsRNA. However, in the RNAi pathway the dsRNA is cleaved into siRNAs, which are the functional units that bind to target message. One longer dsRNA molecule will give rise to more siRNAs than a shorter molecule of dsRNA. Therefore, when calculations are based on weight, the initial number of dsRNA molecules will differ between the 520 bp and 69 bp dsRNA, but the final number of siRNAs should be approximately equivalent.

We questioned whether it is the number of dsRNA molecules introduced or the number of siRNAs produced that determines the RNAi efficiency. If the efficacy of the systemic RNAi response is determined by the efficiency of cellular dsRNA uptake, then the number of dsRNA molecules, rather than the number of siRNAs produced, might be more important. To address this question, we performed a molar dilution series of the 520 bp *EGFP* dsRNA (Figure 2 C) such that the number of initial molecules in each dilution was equivalent to the 69 bp dsRNA dilution series described above (Figure 2 B). In this dilution series, the number of 520 bp and 69 bp dsRNA molecules injected was the same, but there were more siRNAs produced in each of the 520 bp dilutions because the 520 bp fragment is longer than the 69 bp fragment. We found that when a molar dilution of the 520 bp dsRNA was performed there was no EGFP expression in any of the injected individuals at the 100 fold, 1,000 fold, or 10,000 fold dilutions (Figure 2 C1-C3, Table S2). EGFP was not effectively reduced when the 520 bp dsRNA was diluted 100,000 times (Figure 2 C4, Table S2). Since the 10,000 fold dilution of 520 bp dsRNA (Figure 2 C3) but not the 69 bp dsRNA (Figure 2 B3) was able to knock down EGFP, it is not the initial number of dsRNA molecules that determines RNAi efficiency but the number of siRNAs produced. These data suggest that the rate-limiting step affecting the efficiency of the RNAi response is determined by the intracellular RNAi machinery rather than the efficiency of dsRNA uptake.

The data above provide information about the concentration of dsRNA needed to achieve efficient knockdown of EGFP in *Tribolium* wing discs. However, RNAi is not always effective in all tissue types. In *Drosophila,* it is known that RNAi is less effective in wing imaginal tissue [39]. In *C. elegans,* some nervous tissues are refractory to RNAi due to the expression of the nuclease Eri-1, which degrades the siRNA [40]. We have previously shown that virtually all tissues in *Tribolium* larvae and pupae are susceptible to RNAi when high concentrations of dsRNA are used [24]. However, it is possible that not all tissues require the same amount of dsRNA. To determine if any tissues in *Tribolium* require a higher level of dsRNA, we performed a serial dilution of *EGFP* dsRNA (520 bp) and injected it into transgenic beetles in which *EGFP* is driven by the *α-tubulin* promoter causing EGFP expression in all tissues [41] (Figure 2 D). Our data suggest that all tissues in *Tribolium* larvae are similarly susceptible to RNAi, as EGFP expression was effectively reduced in all tissues at a concentration of 0.001 µg/µL in most individuals (1,000 fold dilution) (Figure 2 D1, D2, Table S2).

### Quantification of the dsRNA Size and Concentration Dependency by Quantitative RT-PCR

**Figure 3:**
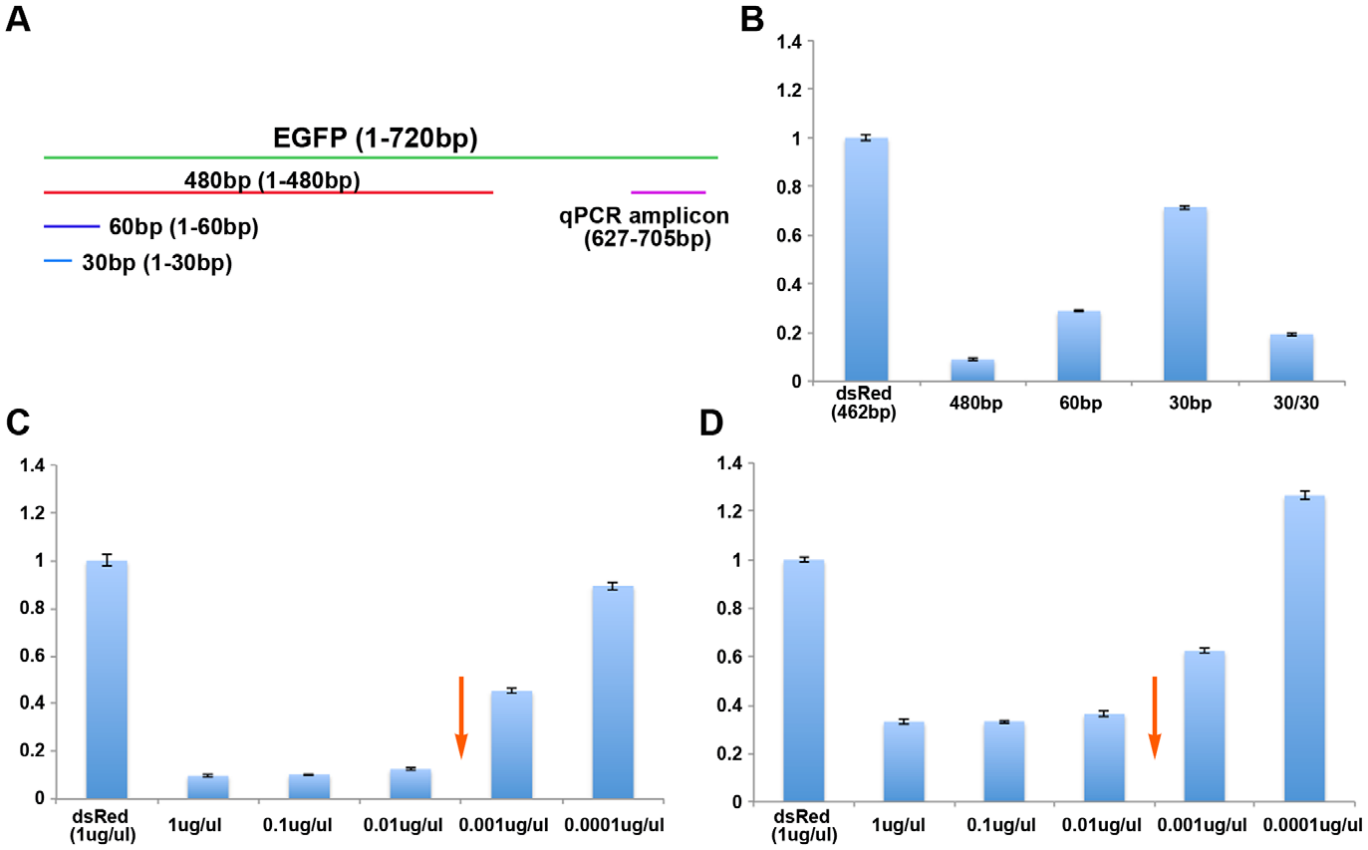
Reduction of *EGFP* mRNA by various sizes and concentrations of EGFP dsRNA. (A) Location of *EGFP* dsRNAs and qPCR amplicon relative to the *EGFP* gene. (B) Reduction of EGFP mRNA induced by various sizes of *EGFP* dsRNA. (C) Reduction of *EGFP* mRNA induced by various concentrations of *EGFP* 480 bp dsRNA. (D) Reduction of *EGFP* mRNA induced by various concentrations of *EGFP* 60 bp dsRNA. Arrows in C and D indicate a potential threshold concentration for inducing efficient RNAi.

To further assess the dsRNA size and concentration dependency in systemic RNAi in *Tribolium*, we have utilized quantitative RT-PCR (qPCR). We designed several new *EGFP* dsRNA molecules (480 bp, 60 bp, and 30 bp) to avoid the qPCR primer site (Figure 3 A). These dsRNA molecules were injected into last instar larvae at the concentration of 1 µg/µL, followed by *EGFP* mRNA quantification five days after injection (Table S3). Among the three lengths of dsRNA tested, 480 bp dsRNA induced the most efficient knock down, exhibiting over 90% reduction of the *EGFP* mRNA level compared to the control dsRed dsRNA injected samples (Figure 3 B). The 60 bp dsRNA induced a moderate level of knock down (70% reduction), while the 30 bp dsRNA was least effective (30% reduction). These data provide quantitative evidence for the dsRNA size dependency in systemic RNAi in *Tribolium*, with a longer dsRNA being more efficient to trigger systemic RNAi.

We also tested a chimeric dsRNA that consists of 30 bp *EGFP* dsRNA (the exact same portion used to make the above 30 bp dsRNA) and an additional 30 bp *Ultrabithorax* (*Ubx*) dsRNA. *Ubx* encodes a transcription factor [42], and does not share any homology with the *EGFP* gene. Therefore, this chimeric dsRNA is longer in length (60 bp) compared to the 30 bp *EGFP* dsRNA, without increasing the sequence variety for the portion important to induce *EGFP* RNAi. The injection of this chimeric dsRNA induced significant reduction of *EGFP* mRNA, reaching over 80% reduction (cf. 30% reduction with 30 bp dsRNA) (Figure 3 B). This result clearly excludes the possibility that the lack of sequence variety caused the short dsRNAs to be ineffective triggers of the RNAi response, since the same 30 bp sequence can trigger efficient RNAi when the length of the dsRNA is increased. Therefore, small dsRNAs are less effective to trigger systemic RNAi, most likely due to inefficient uptake by the cells in *Tribolium*.

The dsRNA concentration dependency in systemic RNAi was also confirmed by qPCR. The serial dilution (from 1 µg/µL) of *EGFP* dsRNA (480 bp) caused gradual reduction of RNAi efficiency (from 90% reduction at 1 µg/µL to 10% reduction at 10,000 fold dilution. Figure 3 C), therefore qPCR provided a better visualization of the dsRNA concentration dependency in the systemic RNAi response. We also noticed that there appears to be a greater reduction of RNAi efficiency between 100 fold and 1,000 fold dilutions compared to other dilutions, which may imply a potential threshold effect in addition to the gradual reduction of RNAi efficiency (arrow in Figure 3 C). The results of the serial dilution using a smaller *EGFP* dsRNA (60 bp) were consistent with those of 480 bp dsRNA. A gradual reduction of RNAi efficiency was observed, albeit less overall RNAi efficiency possibly due to the shorter dsRNA length (Figure 3 D). Taken together, these data provide further evidence that the efficiency of systemic RNAi in *Tribolium* depends on the concentration of initial dsRNA.

It is worth mentioning that the potential threshold point of the 60 bp dsRNA serial dilution (arrow in Figure 3 D) corresponds to that of the 480 bp dsRNA serial dilution (arrow in Figure 3 C), both of them being between 100 fold and 1,000 fold dilutions. As mentioned before, these dilutions were calculated based on dsRNA weight. Therefore, the initial number of dsRNA molecules will differ between the 480 bp and 60 bp dsRNA, but the final number of siRNAs should be approximately equivalent. The fact that the potential threshold points of the 480 bp and 60 bp dsRNA match when calculated based on dsRNA weight gives further evidence that the number of siRNAs produced (and not the initial number of dsRNA molecules provided) determines RNAi efficiency.

### Duration of RNAi Effect

The duration of the RNAi effect varies in organisms that exhibit a systemic RNAi response. The RNAi effect may wear off as the dsRNA is depleted, if dsRNA is not continually expressed within the cell, is not maintained by the cell, or is not continually provided to the organism through continued feeding, soaking, or multiple injections [30], [43]. Several processes have been identified to affect the duration of the RNAi effect. One process is heterochromatin formation via the action of siRNAs. In *C. elegans*, siRNA not only causes post-transcriptional silencing, but also causes transcriptional silencing by making the chromatin status of the target gene inactive [44]. This chromatin modification possibly causes a long-lasting RNAi effect even after the trigger dsRNA is depleted. Another process that might affect the duration of the RNAi effect is the maintenance of dsRNA molecules *in vivo*. Although the molecular mechanism of this process is yet to be determined, it has been shown that the injected dsRNA seems to be maintained even a few weeks after the injection in honeybees [17]. The third mechanism that might affect RNAi duration is the amplification of dsRNA molecules. In plants and *C. elegans,* dsRNA provided to the cell can be amplified via the action of RNA dependent RNA polymerases (RdRPs) [45]–[48]. This amplification mechanism uses mRNA as template to synthesize more dsRNA, thereby increasing the amount of dsRNA available for the RNAi pathway. It has been assumed that an amplification mechanism is needed for organisms that exhibit a prolonged RNAi effect [43]. However, available genome screens have been unable to identify RdRPs in most metazoans, including insects [49], [50]. Therefore, if an amplification method exists in *Tribolium,* it should be via a different mechanism.

Regardless of whether an amplification method exists in *Tribolium,* the RNAi effect appears to be long-lived. While this observation has been made anecdotally, duration has not been quantitatively studied.

**Figure 4:**
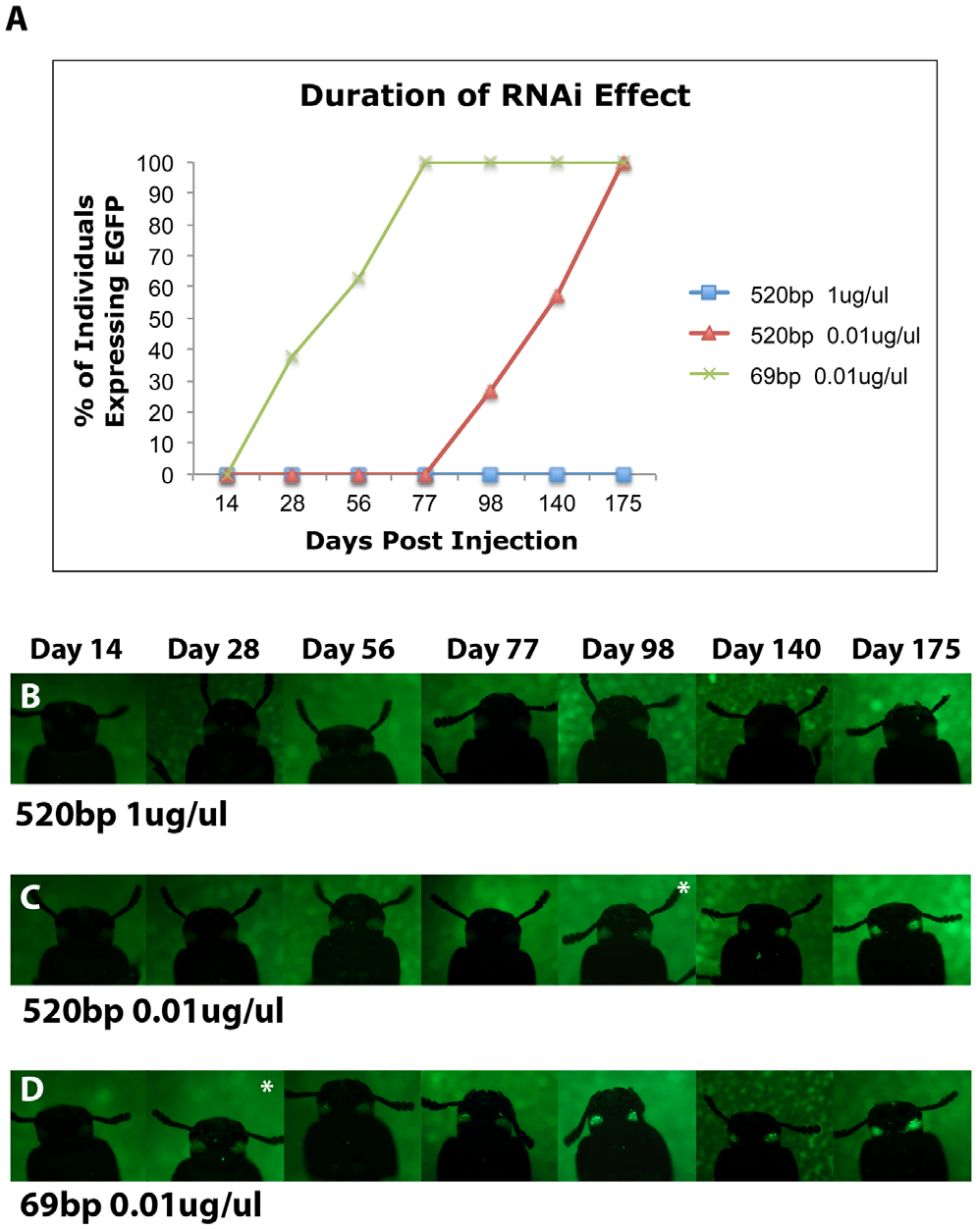
The effect of dsRNA size and concentration on the duration of the RNAi effect. (A) Line graph showing the percentage of individuals expressing EGFP after injection of *EGFP* dsRNA at three different conditions. (B) pu11 *Tribolium* injected with 520 bp *EGFP* dsRNA at a concentration of 1 µg/µL. (C) pu11 *Tribolium* injected with 520 bp *EGFP* dsRNA at a concentration of 0.01 µg/µL. (D) pu11 *Tribolium* injected with 69 bp *EGFP* dsRNA at a concentration of 0.01 µg/µL. Asterisk indicates the first day EGFP expression was detected in the adult eye.

To determine whether concentration influences RNAi duration we injected the 520 bp dsRNA for *EGFP* at two different concentrations (0.01 µg/µL and 1 µg/µL) into pu11 larvae and monitored them weekly for the return of EGFP expression (Figure 4). We monitored EGFP expression in the adult eye instead of wings as the wing EGFP expression fades away after the maturation of wings. At the lower concentration, EGFP expression was first detected in some but not all ommatidia of some individuals 98 days after the injection (n = 15) (Figure 4 A, 4C, Table S4). By day 175 all individuals were showing EGFP expression in some ommatidia (n = 14) (Figure 4 A, 4C, Table S4). At the high concentration EGFP expression did not return in any individuals for the 175 days they were monitored (n = 11) (Figure 4 A, 4B, Table S4). These data suggest that dsRNA concentration does influence the duration of the RNAi effect in *Tribolium*, with higher concentrations of dsRNA being more effective.

To determine whether the size of dsRNA influences the duration of the RNAi effect, we compared individuals in which either the 69 bp or the 520 bp fragment were injected at a concentration of 0.01 µg/µL (which should give rise to approximately the same number of siRNAs) (Figure 4). As mentioned above, when using the 520 bp fragment of dsRNA at a concentration of 0.01 µg/µL, the first individual began to exhibit EGFP expression in some ommatidia on day 98 (n = 15) (Figure 4 A, 4C, Table S4). In contrast, for those individuals injected with the 69 bp fragment at a concentration of 0.01 µg/µL, EGFP expression was first seen in some ommatidia 28 days after injection (n = 8) (Figure 4 A, 4D, Table S4). All individuals in this group expressed EGFP by day 77 (n = 7) (Figure 4 A, 4D, Table S4). These data suggest that size also influences the duration of the RNAi effect in *Tribolium*, with longer fragments increasing the duration.

### dsRNA Competition

Occasionally experiments require the knockdown of multiple genes. In these situations combinatorial delivery of dsRNA can be used to remove the function of multiple genes simultaneously [51]. However, research has shown that oversaturation of the RNAi machinery can occur when multiple dsRNA or siRNAs are delivered [30], [52]–[54]. This oversaturation causes several problems. First, because the miRNA and RNAi pathways share components, oversaturation of these components during the RNAi response can result in unintentional inhibition of the miRNA pathway resulting in phenotypes related to a loss of miRNA function. Because miRNAs are essential for growth, development, and tissue homeostasis, this inhibition may result in lethality [50], [54]. Second, having a mixture of dsRNA can result in competition between the dsRNAs for RNAi machinery components and/or cell entry and transport components resulting in competitive inhibition. This competitive inhibition results in an inability to knock down multiple genes at the same time [55]–[61]. It has been shown that some siRNAs have greater competition potency than others [58], [59], [62]. Therefore, depending on the combination of dsRNAs used different levels of competition may occur.

**Figure 5:**
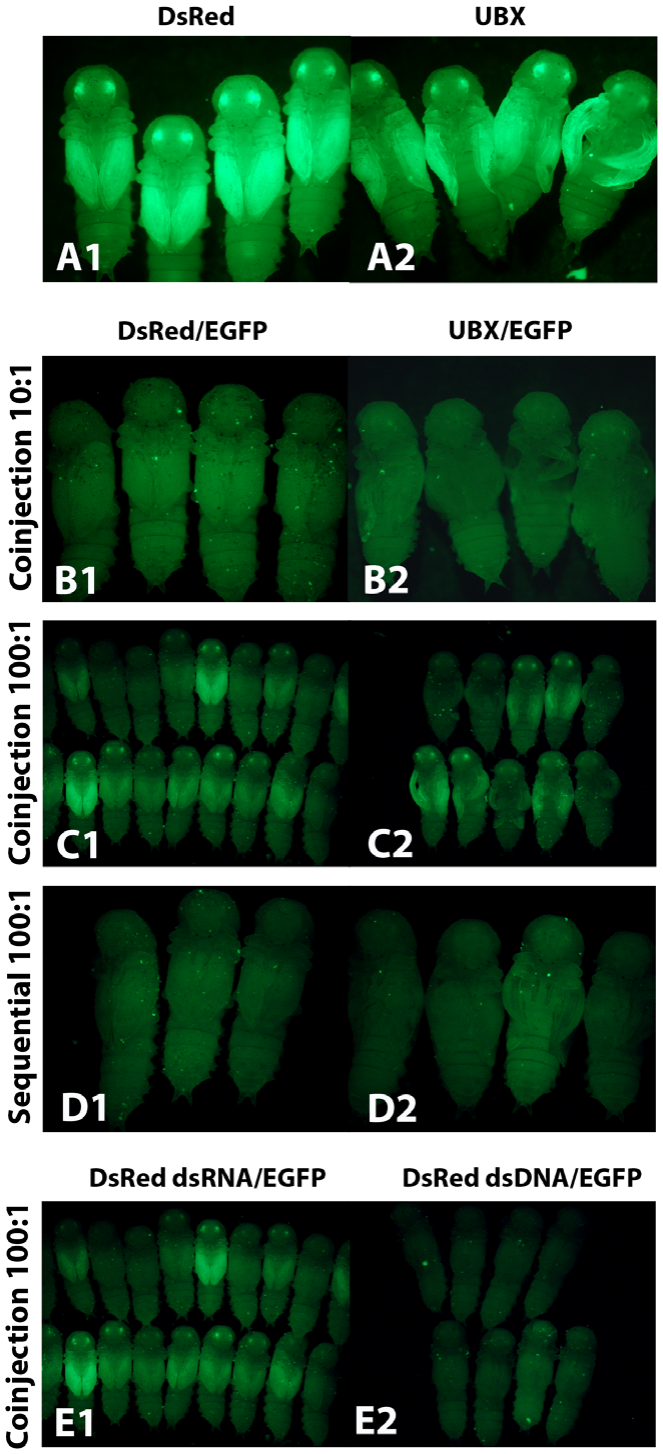
The effect of dsRNA competition on RNAi knockdown efficiency. (A) Competitor dsRNA injected alone. (1) *DsRed* dsRNA. (2) *Ubx* dsRNA. (B) Competitor and reporter dsRNA co-injected at a 10 to 1 ratio. (1) *DsRed* dsRNA injected at 1 µg/µL (competitor). *EGFP* dsRNA injected at 0.1 µg/µL (reporter). (2) *Ubx* dsRNA injected at 1 µg/µL (competitor). *EGFP* dsRNA injected at 0.1 µg/µL (reporter). (C) Competitor and reporter dsRNA co-injected at a 100 to 1 ratio. (1) *DsRed* dsRNA injected at 1 µg/µL (competitor). *EGFP* dsRNA injected at 0.01 µg/µL (reporter). (2) *Ubx* dsRNA injected at 1 µg/µL (competitor). *EGFP* dsRNA injected at 0.01 µg/µL (reporter). (D) Competitor and reporter dsRNA injected sequentially at a 100 to 1 ratio. (1) *DsRed* dsRNA injected at 1 µg/µL (competitor). *EGFP* dsRNA injected at 0.01 µg/µL (reporter). (2) *Ubx* dsRNA injected at 1 µg/µL (competitor). *EGFP* dsRNA injected at 0.01 µg/µL (reporter). (E) *DsRed* dsRNA or *DsRed* dsDNA is used as the competitor nucleic acid. *EGFP* dsRNA is co-injected as the reporter. The ratio of competitor to reporter is 100 to 1. (1) *DsRed* dsRNA injected at 1 µg/µL. *EGFP* dsRNA injected at 0.01 µg/µL. (2) *DsRed* dsDNA injected at 1 µg/µL. *EGFP* dsRNA injected at 0.01 µg/µL.

To determine at what concentration competitive inhibition occurs in *Tribolium*, we injected two dsRNAs simultaneously into pu11 larvae. One dsRNA (the competitor) was used at a higher concentration and one dsRNA (the reporter) was used at a lower concentration. For the competitor we used one of two dsRNAs, *DsRed* or *Ubx* (Figure 5). We chose to use these dsRNAs as competitors for several reasons. First, because some siRNAs may have greater competition potency than others, it is possible that we may see different results by using two different dsRNA competitors. Second, it is possible that having mRNA targets present may affect the competition level. There is no *DsRed* expression in the pu11 beetles, allowing us to test the competition level when the competitor is an exogenous dsRNA with no mRNA target. *Ubx* is expressed in the beetle hindwing but not the forewing [42]. Therefore, in one wing disc the competitor will have a complementary mRNA target and in the other disc it will not. If the presence of target influences the level of competition, we may see differences between the two wing discs. And third, *Ubx* gives a very distinct wing phenotype [42] enabling us to assess whether the *Ubx* competitor is efficient at down regulating *Ubx*. In both cases (*DsRed* and *Ubx* experiments), *EGFP* is used as our reporter gene. By monitoring the EGFP expression in the wing we can determine whether the competitor (*DsRed* or *Ubx*) is preventing the knockdown of EGFP.

When the competitor dsRNA was injected at 10 fold higher concentration than *EGFP* dsRNA, we did not see competition at a level that resulted in inefficient knockdown of EGFP, as EGFP expression was effectively reduced in 100% of individuals (n = 23 and n = 17) (Figure 5 B, Table S5). In contrast, when we injected competitor dsRNA at a 100 fold higher concentration than *EGFP* dsRNA, we did see competition that resulted in inefficient knockdown of EGFP in some individuals (n = 18 and n = 10) (Figure 5 C, Table S5). The same result was seen when either *dsRed* or *Ubx* was used as the competitor, and there was no difference between the EGFP expression in the two wing discs in the *Ubx* experiment. Therefore, our data suggest that the presence of mRNA targets does not affect the competition level.

Competition between dsRNA could occur at several levels. It may occur during cellular uptake and transport of the dsRNA or it may occur during dsRNA processing and mRNA silencing. We reasoned that if the competition is occurring at the level of dsRNA uptake, sequential injection of the competitor dsRNA and the reporter dsRNA may reduce the amount of competition. We injected dsRNA for either *dsRed* or *Ubx*, waited two days, and then injected dsRNA for *EGFP*. When this delay was introduced between injection of the competitor dsRNA and the *EGFP* dsRNA, we no longer saw competition that resulted in inefficient knockdown of EGFP in any individual (n = 11 and n = 19) (Figure 5 D, Table S5), even at the 100 fold higher concentration. These data indicate that sequential injection can decrease the amount of competition that is occurring between dsRNAs, suggesting that competition might be occurring when the dsRNA is entering the cell.

The uptake mechanism of dsRNA in insects is currently unknown. Recently, it has been shown that injected dsDNA can be efficiently taken up by cells and transiently expressed in at least one insect [63]. We hypothesized that the uptake method of dsRNA and dsDNA may be related. If the uptake of all nucleic acids occurs by the same mechanism and if the competition we are seeing is occurring at the uptake level, then dsDNA may be able to compete with dsRNA. To test this hypothesis we co-injected dsDNA for *dsRed* and dsRNA for *EGFP* into pu11 larvae. The dsDNA was at a 100 fold higher concentration than the dsRNA. At this concentration level when two dsRNAs were coinjected there is competition (Figure 5 E1, Table S5), resulting in inefficient EGFP knockdown. In contrast, when dsDNA was used, we did not see competition in any of the injected individuals (n = 29) (Figure 5 E2, Table S5). These results suggest that either the transport mechanism of dsRNA is RNA specific or the competition is occurring at a different level.

## Discussion

Within the last decade, RNAi has become a genetic tool that has made the functional study of genes in non-model systems readily available. While there has been a race to identify the next organism in which RNAi can be used, the details have often been overlooked. Given the obvious variety of mechanisms underlying RNAi, the optimal condition to achieve efficient RNAi likely varies among species. The work described here provides specific data on the essential parameters for RNAi in the red flour beetle, and also encourages researchers to investigate these parameters when planning RNAi experiments in other organisms.

### dsRNA Size and Systemic RNAi in *Tribolium*

With regard to dsRNA size, we established that long dsRNA appears to be the most effective with respect to both the initial knockdown and the duration of the RNAi effect. While both a 69 bp and a 520 bp dsRNA were capable of resulting in gene knockdown, the 520 bp fragment was more effective. Our qPCR studies also provided evidence that long dsRNA is more effective. Several explanations are possible for why the 69 bp dsRNA was less efficient. First, while the 69 bp dsRNA will give rise to the same number of siRNAs (when compared to an equal weight of 520 bp dsRNA), the types of siRNAs produced will be more limited with regard to sequence. Therefore, the possibility exists that the longer dsRNA is not more effective because it is longer but because it produces a greater variety of siRNAs some of which may be more effective at silencing than the limited number of siRNAs produced by the shorter dsRNA fragment. Another possibility is that while the 69 bp dsRNA is most assuredly taken up by the cell, the efficiency of this uptake may not be as high, resulting in a lower quantity of siRNAs available for silencing. Finally, if a dsRNA amplification or storage mechanism occurs in *Tribolium*, these processes may also be affected by dsRNA size.

While dsRNA 60 bp and longer did result in gene knockdown, smaller dsRNAs (30, 31, and 21 bp) were ineffective at gene silencing when injected into *Tribolium* at a multicellular stage. Our data provide clear evidence that this is due to inefficient uptake of the shorter dsRNA fragments. Data from *C. elegans* and *Drosophila* S2 cells support this hypothesis [10], [28]–[31]. Interestingly, the use of siRNAs has effectively achieved gene knockdown in the pea aphid [34], the termite [35], and the spider mite [64], which might suggest that insect cells from different species may recognize and/or uptake dsRNA in different ways. An alternative explanation is that the siRNA samples contained a mixture of long dsRNA and siRNAs, as siRNAs were derived from long dsRNA cleaved *in vitro* by the Dicer enzyme in these experiment [34], [35].

We have previously reported that even the 31 bp dsRNA was capable of triggering a systemic RNAi response in *Tribolium* [15]. However, we found that this was due to contamination of longer dsRNA molecules. While making template DNA by PCR for dsRNA synthesis, we noticed that a primer we used bound to a secondary site, producing a longer template DNA than we intended. Sequential dsRNA synthesis utilizing this template DNA produced dsRNA molecules longer than 50 bp in addition to the intended 31 bp molecules. We used a *de novo* synthesized DNA template to avoid this problem in this study (see Materials and Methods), and obtained a different result (corrigendum will be submitted).

### Duration of the Systemic RNAi Response in *Tribolium*

We observed that dsRNA size seems to have a more drastic affect on RNAi duration than on the initial RNAi efficiency. For example, when the 69 bp fragment was used at a concentration of 0.01 µg/µL the first individual began to express EGFP 28 days post injection. In contrast, it took 98 days with 520 bp dsRNAs at the same concentration. This result may suggest a length-dependent dsRNA maintenance or amplification mechanism in beetles.

In *C. elegans*, amplification by RdRP is an essential step to achieve any RNAi effect [46], [47]. Therefore, it has been assumed that an amplification mechanism is needed in all organisms that exhibit a prolonged RNAi effect. However, not only have RdRPs not been found in insects [50], but it has also been shown that isoform specific RNAi can be performed in *Tribolium* [65], suggesting that amplification using the endogenous mRNA as template is not occurring. There remains a possibility that amplification is occurring through another mechanism (perhaps using the dsRNA as template, as this would still allow for isoform specific RNAi). However, amplification may not be needed to achieve an effective long-lasting RNAi effect. It is possible that insects with a robust systemic RNAi effect simply have the ability to efficiently take up and/or store dsRNA. In *Tribolium*, RNAi efficiency appears to correlate well with the initial dose of dsRNA molecules injected (i.e. the amount of initially injected dsRNA molecules determines the degree of RNAi effect. See Supplemental Figure 4 in [66] for example). Dose-dependent RNAi is less likely to be observed if the initial RNAi triggers are amplified, also supporting the idea that there is no robust amplification mechanism in *Tribolium*.

While the mechanism of RNAi duration in *Tribolium* has not been determined, we were able to show that when dsRNA is injected at the last larval stage the effect can last for many months, perhaps even for the entire lifespan of the individual. It has also been shown that parental RNAi (in which female pupae or adults are injected with dsRNA and the effect is seen in the offspring) can also be effective for several months [14], suggesting an extremely efficient RNAi response. However, parental RNAi appears to be less efficient when last instar larvae are injected with dsRNA (data not shown. Generality of this tendency requires further validation). One explanation for this is that the female reproductive organs do not complete formation until the pupal stage. Perhaps in order for the oocytes to efficiently uptake dsRNA they must be formed at the time of dsRNA introduction to the body cavity. If this is true, we might expect to see the RNAi effect lasting longer in established tissue and being less effective in tissue that is continually turned over.

In *C. elegans*, RNAi can sometimes last more than one generation through chromatin remodeling [44], [67]. The long lasting RNAi effect in *Tribolium* could infer a similar type of epigenetic controls. However, the RNAi effect in *Tribolium* does not appear to be permanent since EGFP expression eventually returned after *EGFP* dsRNA injection, suggesting that the long-lasting RNAi effect in *Tribolium* is not caused by epigenetic control.

In addition to data concerning the effect of dsRNA size and concentration on the duration of RNAi, the duration experiments revealed several other interesting observations. When the RNAi effect wears off in the adult, EGFP expression appears to return one ommatidia at a time in a mosaic pattern across the surface of the eye. In a process that takes weeks (or even months), more and more ommatidia begin to express EGFP until EGFP in the eye reaches wildtype levels. The pattern of EGFP return suggests that, at least in the eye, the RNAi is acting cell autonomously. Additionally, we also observed that there were vast differences in the length of time it took for EGFP to return in one experimental group. For example, when the 69 bp fragment was used at a concentration of 0.01 µg/µL the first individual began to express EGFP 28 days post injection. However, EGFP was not seen in all individuals until 77 days post injection. These differences probably represent subtle differences in injection volume between individuals, but may also reflect variation in the injection site (distance from the eye) or in an individual beetle’s ability to uptake, store, or amplify the dsRNA.

### Applicability to Other Genes

It is important to note that with regard to the parameters tested in this study the results are probably gene specific. For example, EGFP expression was removed with a concentration of only 0.001 µg/µL, which may not be efficient to deplete the expression of other genes. In our labs, RNAi experiments that are intended to result in complete gene knockdown are generally performed with dsRNA concentrations varying between 1 and 4 µg/µL. We have previously shown that *Ubx* RNAi at the larval stage also shows dsRNA concentration dependency in *Tribolium* [66]. In addition, we tested several different sizes of dsRNA molecules for *Ubx* (480 bp, 60 bp and 30 bp) and saw that the efficiency of *Ubx* RNAi also depends on the length of dsRNA (data not shown). Hence, while the exact dsRNA size and concentration required and the exact number of days an effect will last will probably vary between genes, we expect the trends to remain the same; longer dsRNA and higher concentrations of dsRNA should result in more efficient gene knockdown and a longer knockdown duration.

In this study, we focused our analysis on the expression of *EGFP*. The exogenous nature of the *EGFP* gene provides us with a less biased view of how parameters relating to dsRNA molecules affect RNAi efficiency, as the expression of EGFP is not influenced by complex endogenous gene regulatory networks (such as positive or negative feedbacks) or other post-transcriptional regulations (such as alternative splicing).

Therefore, the outcomes of our study for parameters affecting the efficiency of RNAi by using *EGFP* as a model are likely to be applicable to other genes.

### Competition between dsRNAs in *Tribolium*

Because the uptake, transport, and processing of dsRNA all require cellular components and proteins that are finite, competition for these components will occur at some level. The question is, which level is most sensitive to oversaturation. Once dsRNA is injected into the individual, dsRNA that is not taken up into cells is presumably excreted. Therefore, the uptake of dsRNA must occur relatively quickly. However, the duration of the RNAi effect suggests that mRNA silencing occurs over an extended period of time. Therefore, the fact that sequential injection of multiple dsRNA appears to lower the level of competition suggests that the competition seen in our assay is occurring at the level of dsRNA uptake (although competition at the mechanism level may also occur at particular ratios). Regardless of the step at which competition is occurring, our data indicate that at certain ratios, combinatorial delivery of dsRNA can result in competitive inhibition. As competition potency may vary between dsRNAs, this ratio may vary depending on the combination of dsRNAs used.

Our competition experiments did not show any observable impact on the miRNA pathway. miRNAs are essential for growth and development, and are specifically known to be required for metamorphosis in insects [68]. Additionally, we have shown that knockdown of Tc-Argonaute-1 (an essential component of the miRNA machinery) results in larval lethality [50]. However, we never saw any lethality or developmental phenotypes indicating that the miRNA pathway was impaired due to oversaturation of miRNA pathway components. Higher concentrations of dsRNA may be required to inhibit the miRNA pathway in *Tribolium*. It may also be possible that miRNA inhibition by oversaturation of the RNAi machinery may be more difficult to achieve in *Tribolium* due to subfunctionalization of the machinery components. Both *Drosophila* and *Tribolium* appear to have proteins, such as Argonaute-1 and Argonatue-2, that have duplicated and subfunctionalized such that one protein is involved in the miRNA pathway while the other is involved in the RNAi pathway [69]. Therefore, it is possible that in these insects accidental inhibition of the miRNA pathway may occur less often.

## Methods

### Beetle Strains

Two transgenic lines of beetles were used in these studies. pu11 beetles [15], [36] are an enhancer trap line in which an EGFP variant is expressed in the nervous system of first instar larvae, in the eyes and wing discs of last instar larvae, in the eyes and wings of pupae, and in adult eyes. Although this line is published as an enhancer trap line that expresses EGFP [36], we recently found that it actually expresses one of the EGFP variants (EYFP) instead of EGFP. There are only seven nucleotide differences between *EGFP* and *EYFP* genes (Figure S1), and we confirmed that dsRNA for *EGFP* is as efficient as dsRNA for EYFP to knock down EYFP in pu11 (data not shown). AT^11^ is a transgenic line in which *EGFP* is driven by an *α-tubulin* promoter [41]. In the AT^11^ line, EGFP is expressed ubiquitously at all life stages.

### dsRNA Synthesis

Template preparation for dsRNA synthesis of *Ubx* and *DsRed* for the competition experiment was performed by PCR. The primer was designed against the pCR4-TOPO vector sequence flanking the insertion site, with a T7 promoter sequence at the 5′ end as described previously [50], [70]. Template preparation for longer *EGFP* dsRNA fragments (520 bp, 480 bp, 69 bp and 60 bp), as well as for 462 bp *dsRed* dsRNA, was performed by PCR using template gene specific forward and reverse primers with a T7 promoter sequence at their 5′ ends (Table 1 and 2). For the shorter *EGFP* dsRNA fragments (30 bp and 31 bp) *EGFP* template was not used. Instead overlapping primers corresponding to a small region of the *EGFP* coding region were designed with a T7 promoter sequence at their 5′ ends (Table 1). These overlapping primers were dimerized in a PCR reaction mix at 50°C for 20 minutes. For the 30 bp *EGFP* dsRNA used for the qPCR study, we used *de novo* synthesized oligos to produce the dsRNA template (Table 2). The sense and anti-sense oligos corresponding to the first 30 bp region of the *EGFP* coding region with a T7 promoter sequence at their 5′ ends were annealed to produce the double-stranded DNA template for dsRNA synthesis (95°C for 30 minutes then left for 1 hour at room temperature for annealing). These *de novo* synthesis methods were chosen to avoid the possibility of synthesizing longer dsRNA than intended due to primers binding to the template in a non-specific manner. We also used *de novo* synthesized oligos to produce the *EGFP-Ubx* chimeric template (Table 2). Because of the length of these chimeric oligos, we used IDT Ultremer oligo service to secure the accuracy. dsRNA was synthesized using the MEGAscript T7 High Yield Transcript kit (Ambion). Silencer GFP (eGFP) siRNA (21 bp) was purchased (Ambion). Information regarding the exact sequences and the target sites of this product is confidential, and we were unable to obtain any sequence information from the company. However, Ambion responded to our inquiry, and stated that this product does target the *EGFP* gene we used in this study. The positive result of our embryonic injection also provides evidence that this product is an efficient *EGFP* RNAi trigger when introduced directly inside the cell.

**Table 1:** Primers used to synthesize *EGFP* dsRNA templates.

Primer	Sequence	dsRNA size
GFPiF2	TAATACGACTCACTATA **G**GG CGATGCCACCT	520 bp
GFPiR5	TAATACGACTCACTATA **G**GG CGGACTGGGTG	
GFPiF2	TAATACGACTCACTATA **G**GG CGATGCCACCT	69 bp
GFPiR2	TAATACGACTCACTATA **G**GG CACGGGCAGCT	
GFPiF1d	TAATACGACTCACTATA **G**GG CGATGCCACCTACGGCAAG	31 bp
GFPIR1d	TAATACGACTCACTATA **G**GG TCAGCTTGCCGTAGGTGGC	
GFPiF1d2	TAATACGACTCACTATA **G**GG ATCTGCACCACCGGCAAGCTGCC	31 bp
GFPiR1d2	TAATACGACTCACTATA **G**GG AGGGCACGGGCAGCTTGCCGGTG	
FragF2	TAATACGACTCACTATA **G**GG CACCTACGGCAAGCTGACCCTGA	30 bp
FragR2	TAATACGACTCACTATA **G**GG ATGAACTTCAGGGTCAGCTTGC	
FragF3	TAATACGACTCACTATA **G**GG AAGCTGACCCTGAAGTTCATCTG	30 bp
FragR3	TAATACGACTCACTATA **G**GG GTGGTGCAGATGAACTTCAGG	
FragF4	TAATACGACTCACTATA **G**GG TGAAGTTCATCTGCACCACCGGC	30 bp
FragR4	TAATACGACTCACTATA **G**GG CAGCTTGCCGGTGGTGCAGAT	
FragF5	TAATACGACTCACTATA **G**GG CTGCACCACCGGCAAGCTGCCCG	30 bp
FragR5	TAATACGACTCACTATA **G**GG CAGGGCACGGGCAGCTTGCCGG	
FragF6	TAATACGACTCACTATA **G**GG GCAAGCTGCCCGTGCCCTGGCC	30 bp
FragR6	TAATACGACTCACTATA **G**GG AGGGTGGGCCAGGGCACGGGC	
FragF7	TAATACGACTCACTATA **G**GG CCGTGCCCTGGCCCACCCTCGTG	30 bp
FragR7	TAATACGACTCACTATA **G**GG GGTGGTCACGAGGGTGGGCCA	
FragF8	TAATACGACTCACTATA **G**GG CCCACCCTCGTGACCACCCTGA	30 bp
FragR8	TAATACGACTCACTATA **G**GG CCGTAGGTCAGGGTGGTCACGA	

Underlined represents the minimum promoter sequence for T7 polymerase.

Bold **G** is the first based incorporated into RNA during transcription.

**Table 2:** Primers and oligos used to synthesize *EGFP* and *dsRed* dsRNA templates for qPCR analysis.

Primer	Sequence	dsRNA size
EGFPi New F1	TAATACGACTCACTATA **G**GG ATGGTGAGCAAGGGC	480 bp
EGFPi New R1	TAATACGACTCACTATA **G**GG GTTCTTCTGCTTGTC	
EGFPi New F1	TAATACGACTCACTATA**G**GG ATGGTGAGCAAGGGC	60 bp
EGFPi New R2	TAATACGACTCACTATA**G**GG GTCCAGCTCGACCAG	
EGFP NEW 30 F1	TAATACGACTCACTATA**G**GG ATGGTGAGCAAGGGCGAGGAGCTGTTCACC	30 bp
	CC**C**TATAGTGAGTCGTATTA	
EGFP NEW 30 R1	TAATACGACTCACTATA**G**GG GGTGAACAGCTCCTCGCCCTTGCTCACCAT	
	CC**C**TATAGTGAGTCGTATTA	
DSREDi F1	TAATACGACTCACTATA**G**GG GACATCCCCGACTAC	462 bp
DSREDi R1	TAATACGACTCACTATA**G**GG TCTGTGCCTGCTCTT	
EGFP-UBX 30 F1	TAATACGACTCACTATA**G**GG ATGGTGAGCAAGGGCGAGGAGCTGTTCACC	30/30 bp
	GCCTACCGCTCCTTCCCGCTGTCGCTCGGCCC**C**TATAGTGAGTCGTATTA	
EGFP-UBX 30 R1	TAATACGACTCACTATA**G**GG GCCGAGCGACAGCGGGAAGGAGCGGTAGG	
	CGGTGAACAGCTCCTCGCCCTTGCTCACCATCC**C**TATAGTGAGTCGTATTA	

Underlined represents the minimum promoter sequence for T7 polymerase.

Bold **G** (or **C** on the antisense oligos) is the first based incorporated into RNA during transcription.

### Injection

Larvae were injected as described previously [15], [70]. For each experimental condition, 20–40 last instar larvae were injected from one dsRNA preparation (see supplemental tables for exact numbers and survival rates). The exact stage was determined by the size of EGFP positive wing discs. We selected larvae that had the initial stage of EGFP expression in the wing disc, which corresponds to 2.5 days after the final larval molt. dsRNA was injected into the dorsal side of the first abdominal segment (A1) at a concentration of 1 µg/µL unless otherwise stated. Each larva can hold up to 0.7 µL of solution when injected at the stage descried above. The volume injected in each larva was calculated by dividing the total volume of dsRNA solution used in one round of injection by the number of larvae injected (e.g. if 8 µL was used for 12 larvae, the average volume injected into each larva was 0.67 µL). With our injection setting, we constantly inject 0.6 ul into each larva (∼0.6 µg/larva with 1 µg/µL dsRNA solution). See [70] for more information. Co-injection of the eight fragments of 30 bp dsRNA was performed at a total concentration of 1 µg/µL (therefore the concentration of each 30 bp dsRNA was 0.125 µg/µL). Competition experiments sometimes involved two separate injections. The second injection was performed 48 hours after the first injection. Molar dilutions were determined by calculating the molecular weight of dsRNA. For these calculations it was assumed that each of the nucleotide bases were equally represented (therefore 1,000 bp of dsRNA 0.73 µg = 1 pmol). After injection, larvae were maintained on culture flour at 30°C.

### EGFP Fluorescence Analysis

In the first instar larvae, we focused our analysis on the nervous system EGFP expression because of its stable and robust expression level. For the analysis in the last larval stage, we focused on the wing disc EGFP expression. We excluded the nervous system expression during the last larval stage from our analysis because the EGFP intensity in the nervous system in the pu11 beetle during the last larval instar is unstable and oscillates. We also did not score larval eye EGFP expression, as EGFP fluorescence in the eye appears to be insensitive to RNAi at the last larval stage. This is possibly because larval eyes are already formed and have a decent amount of EGFP proteins at the time of injection. Therefore, EGFP protein perdurance and/or lack of cell proliferation in the matured larval eye possibly mask the effect of *EGFP* RNAi for several days after injection. For the analysis at the adult stage, we monitored EGFP expression in the adult eye instead of wings as the wing EGFP expression fades away due to cell death after the maturation of wings.

For larval documentation, larvae were sifted from the flour and submerged in water, which causes the larvae to stop moving, and, if they are removed from the water within several hours, is not lethal. After documentation, the larvae were removed from the water and dried briefly on tissue paper before being returned to the culture flour. Larvae were monitored for fluorescence five days after the initial injection using a filter set capable of detecting both EGFP and EYFP. Pupae were documented 12 days after the initial larval injection. For the duration experiment, adults were documented weekly. Larvae, pupae, and adults were documented using an Olympus SZX12 microscope equipped with a Nikon DXM 1200F digital camera. Identical exposure times were used for all the images in the same experiment.

### Quantitative RT-PCR

Total RNA was isolated from pupae 5 days after injection (two pupae per sample) using Maxwell 16 automated RNA isolation system with Maxwell 16 LEV simplyRNA Purification Kit (Promega). The quality of RNA was validated by Agilent Bioanalyzer. cDNA was synthesized using iScript Reverse Transcription Supermix for RT-qPCR (Bio-Rad). Quantitative real-time PCR was performed by using SsoAdvanced SYBR Green Supermix with CFX Connect Real-Time PCR Detection System (Bio-Rad). Two genes, ribosomal protein gene *RPS3e* and *RPL13a*, were used as reference for ΔΔCq quantification. Primer sequences for the reference genes as well as for *EGFP* are listed in Table 3. The amplification efficiency of these primers was determined by using serially diluted cDNA samples (Table 3). All PCR was run as triplicate and the data were analyzed by CFX Manager.

**Table 3:** Primers used for qPCR analysis.

Primer	Sequence	Amplicon	efficiency
RP S3Eq F1	TTTGTATGGCGAGAAGGTGG	117 bp	82.70%
RP S3Eq R1	CTCAAAACACCATAGCAAGCC		
RP L13Aq F1	ACAAGACAGAACGTGGGAAG	80 bp	80.90%
RP L13Aq R1	TTTCCTGCGGTCATATGGTG		
EGFPq F3	AAAGACCCCAACGAGAAGC	78 bp	85.80%
EGFPq R3	GTCCATGCCGAGAGTGATC		

## Supporting Information

Figure S1 **A sequence comparison between** ***EGFP*** **and** ***EYFP*** **.** There are seven nucleotide differences between these *EGFP* genes (blue shaded). The 520 bp *EGFP* dsRNA, but not the 69 bp dsRNA, contains the location that varies between *EGFP* and *EYFP* (the locations of the primers used to synthesize dsRNA in this study are indicated by red arrows).
(PDF)

Table S1 **dsRNA size requirements.**
(PDF)

Table S2 **dsRNA concentration requirements.**
(PDF)

Table S3 **quantitative real-time PCR assays.**
(PDF)

Table S4 **Duration assays.**
(PDF)

Table S5 **Competition assays.**
(PDF)
