# Supplementary material for: Dissecting Systemic RNA Interference in the Red Flour Beetle Tribolium castaneum: Parameters Affecting the Efficiency of RNAi
Source: PLoS One. 2012 Oct 25;7(10):e47431. doi: 10.1371/journal.pone.0047431 (PMC3484993; doi:10.1371/journal.pone.0047431)
Supplement: Table S1 — dsRNA size requirements. (PDF) [file pone.0047431.s002.pdf]

Table S1: dsRNA size requirements

| Treatment | #Injected | #Surviving | #GFP+ | %GFP+ |
|---|---|---|---|---|
| 520bp-larvae | 32 | 29 | 0 | 0 |
| 69bp-larvae | 25 | 8 | 0 | 0 |
| siRNA-larvae | 40 | 28 | 28 | 100 |
| 31bp-larvae | 27 | 20 | 20 | 100 |
| 2-31bp-larvae | 24 | 19 | 19 | 100 |
| 8-~30bp-larvae | 26 | 21 | 21 | 100 |
| 8-~30bp-eggs | ~300 | 16 | 2 | 11 |
| 520bp-eggs | ~225 | 13 | 0 | 0 |
