# Supplementary material for: Dissecting Systemic RNA Interference in the Red Flour Beetle Tribolium castaneum: Parameters Affecting the Efficiency of RNAi
Source: PLoS One. 2012 Oct 25;7(10):e47431. doi: 10.1371/journal.pone.0047431 (PMC3484993; doi:10.1371/journal.pone.0047431)
Supplement: Table S2 — dsRNA concentration requirements. (PDF) [file pone.0047431.s003.pdf]

Table S2: dsRNA concentration requirements

Treatment	Strain	#Injected	#Surviving	#GFP+	%GFP+
520bp-0.01ug/ul	Pu11	30	26	0	0
520bp-0.001ug/ul	Pu11	29	23	0	0
520bp-0.0001ug/ul	Pu11	29	25	19	76
520bp-0.00001ug/ul	Pu11	24	19	19	100
69bp-0.01ug/ul	Pu11	26	15	2	13
69bp-0.001ug/ul	Pu11	27	18	2	11
69bp-0.0001ug/ul	Pu11	27	17	17	100
69bp-0.00001ug/ul	Pu11	27	16	16	100
520bp-100molar dilution	Pu11	34	31	0	0
520bp-1000molar dilution	Pu11	23	18	0	0
520bp-10000molar dilution	Pu11	29	20	0	0
520bp-100000molar dilution	Pu11	26	21	21	100
520bp-0.01ug/ul	α tubulin	43	20	0	0
520bp-0.001ug/ul	α tubulin	41	15	4	27
520bp-0.0001ug/ul	α tubulin	35	16	16	100
520bp-0.00001ug/ul	α tubulin	30	11	11	100
