# Supplementary material for: Dissecting Systemic RNA Interference in the Red Flour Beetle Tribolium castaneum: Parameters Affecting the Efficiency of RNAi
Source: PLoS One. 2012 Oct 25;7(10):e47431. doi: 10.1371/journal.pone.0047431 (PMC3484993; doi:10.1371/journal.pone.0047431)
Supplement: Table S3 — quantitative real-time PCR assays. (PDF) [file pone.0047431.s004.pdf]

Table S3: quantitative real-time PCR analysis

dsRNA injected	conc	# injected	# surving	total dsRNA solution used (ul)	amount/larva (ug)	total RNA from 2 pupae (ug/ul)
dsRed	1ug/ul	12	12	8	0.67	225.23
EGFP480	1ug/ul	11	11	8	0.73	232
EGFP480	100ng/ul	6	6	4	0.66	211
EGFP480	10ng/ul	6	6	4	0.66	225.68
EGFP480	1ng/ul	6	6	4	0.66	221.72
EGFP480	100pg/ul	6	6	4	0.66	224.19
EGFP480	10pg/ul	6	6	4	0.66	207.74
EGFP60	1ug/ul	10	10	7	0.70	221.21
EGFP60	100ng/ul	6	6	4	0.67	212.2
EGFP60	10ng/ul	5	5	3	0.60	225.6
EGFP60	1ng/ul	6	6	3.5	0.58	218.9
EGFP60	100pg/ul	6	6	4	0.67	229.3
EGFP60	10pg/ul	6	6	4	0.67	221.5
EGFP30	1ug/ul	10	10	7	0.70	247.36
EGFP-Ubx 30-30	1ug/ul	10	10	7	0.70	240.94
