# Supplementary material for: Dissecting Systemic RNA Interference in the Red Flour Beetle Tribolium castaneum: Parameters Affecting the Efficiency of RNAi
Source: PLoS One. 2012 Oct 25;7(10):e47431. doi: 10.1371/journal.pone.0047431 (PMC3484993; doi:10.1371/journal.pone.0047431)
Supplement: Table S4 — Duration assays. (PDF) [file pone.0047431.s005.pdf]

Table S4: Duration assays

Treatment	# Injected	GFP+/total Day 14	GFP+/total Day 28	GFP+/total Day 56	GFP+/total Day 77	GFP+/total Day 98	GFP+/total Day 140	GFP+/total Day 175
520bp1ug/ul	26	0/13	0/11	0/11	0/11	0/11	0/11	0/11
520bp0.01ug/ul	24	0/15	0/15	0/15	0/15	4/15	8/14	14/14
69bp0.01ug/ul	24	0/10	3/8	5/8	7/7	5/5	5/5	5/5
