# Supplementary material for: Dissecting Systemic RNA Interference in the Red Flour Beetle Tribolium castaneum: Parameters Affecting the Efficiency of RNAi
Source: PLoS One. 2012 Oct 25;7(10):e47431. doi: 10.1371/journal.pone.0047431 (PMC3484993; doi:10.1371/journal.pone.0047431)
Supplement: Table S5 — Competition assays. (PDF) [file pone.0047431.s006.pdf]

Table S5: Competition assays

Treatment	#Injected		#Surviving	#GFP+	%GFP+
dsRed/EGFP 10:1	23		23	0	0
Ubx/EGFP 10:1	17		17	0	0
dsRed/EGFP 100:1	34		18	8	44
Ubx/EGFP 100:1	15		10	6	60
dsRed DNA/EGFP 100:1	34		29	0	0

Treatment	Injection 1	Injection 2 (EGFP)	#Surviving	#GFP+	%GFP+
Sequential dsRed/EGFP 100:1	42	11	11	0	0
Sequential Ubx/EGFP 100:1	51	19	19	0	0
